# A Noisy Analog-to-Digital Converter Connects Cytosolic Calcium Bursts to Transcription Factor Nuclear Localization Pulses in Yeast

**DOI:** 10.1534/g3.118.200841

**Published:** 2018-12-20

**Authors:** Ian S. Hsu, Bob Strome, Sergey Plotnikov, Alan M. Moses

**Affiliations:** Department of Cell & Systems Biology, University of Toronto, Toronto ON, Canada M5S 3B2

**Keywords:** stochastic pulsing transcription factor, Crz1, time delay model, mathematical modelling, calcineurin pathway

## Abstract

Several examples of transcription factors that show stochastic, unsynchronized pulses of nuclear localization have been described. Here we show that under constant calcium stress, nuclear localization pulses of the transcription factor Crz1 follow stochastic variations in cytosolic calcium concentration. We find that the size of the stochastic calcium bursts is positively correlated with the number of subsequent Crz1 pulses. Based on our observations, we propose a simple stochastic model of how the signaling pathway converts a constant external calcium concentration into a digital number of Crz1 pulses in the nucleus, due to the time delay from nuclear transport and the stochastic decoherence of individual Crz1 molecule dynamics. We find support for several additional predictions of the model and suggest that stochastic input to nuclear transport may produce noisy digital responses to analog signals in other signaling systems.

Cells transmit information through signaling pathways. Rather than simple “ON” or “OFF” responses, several key pathways (p53, NF-κB, and others) are now appreciated to encode information in the dynamics of the signaling response (Cai *et al.* 2008; Ashall *et al.* 2009; Locke *et al.* 2011; Purvis *et al.* 2012; Albeck *et al.* 2013; Levine *et al.* 2013; Hansen and O’Shea 2015, 2016; Nandagopal *et al.* 2018). Here we focus on the calcium signaling pathway in yeast, which controls gene transcription through frequency modulation (FM) of the transcription factor Crz1 (Cai *et al.* 2008). In this system, the analog external calcium concentration is converted into the frequency of digital pulses of nuclear localization (discrete rapid rising and falling of nuclear concentration on the order a few minutes). Pulsing is hypothesized to be important in coordinating expression of a large number of genes (Cai *et al.* 2008), increasing amount of information transmitted through the signaling pathway (Levine *et al.* 2013; Hansen and O’Shea 2015), and constructing regulatory logic with other pulsing transcription factors (Lin *et al.* 2015; AkhavanAghdam *et al.* 2016). Understanding the mechanisms of pulsatility is important to understand how information is processed in cells.

Because single-cell observations of signaling pathway activity upstream of pulsing transcription factors are rarely possible, in general, the connection between second messengers and pulsing transcription factors remains unclear. Mechanistic models of FM pulsatile transcription factors have relied on negative feedback coupled with positive feedback (Geva-Zatorsky *et al.* 2006; Locke *et al.* 2011; Albeck *et al.* 2013; Jiang *et al.* 2017) or delayed negative feedback(Geva-Zatorsky *et al.* 2006; Longo *et al.* 2013) to connect pulsatile dynamics to the upstream activity. However, whether there is a negative feedback loop in the calcium signaling pathway that can generate Crz1 pulses is unclear (discussed further below). Furthermore, in each cell, during each Crz1 nuclear localization pulse, approximately 500 Crz1 molecules are transported in and out of the nucleus in a coordinated fashion(Kulak *et al.* 2014), but these pulses are stochastic (and not synchronized between cells). To our knowledge, no mechanistic model of this process has yet been proposed.

One possibility is that Crz1 nuclear localization pulses are connected to variation in cytosolic calcium concentration ([Ca^2+^]_cyt_) since calcineurin is activated by calcium ions and is known to regulate Crz1 localization directly (Cyert 2003). The calcium/calcineurin pathway is widely conserved in species including mammals and yeasts (Goldman *et al.* 2014). In fungi, calcineurin is a phosphatase that activates stress responses and maintains drug resistance through protein-protein interaction or genetic interaction via Crz1, which activates more than 100 genes(Yoshimoto *et al.* 2002 p. 1; Goldman *et al.* 2014; Juvvadi *et al.* 2014). Calcium bursts have been observed in many cell types, including yeast(Tsien and Tsien 1990; Tian *et al.* 2009; Song *et al.* 2012; Sneyd *et al.* 2017; Carbó *et al.* 2017), but the connections between cytosolic calcium concentration and Crz1 localization have not been analyzed in single cells. Mechanistic models of Crz1 regulation through calcium signaling do not predict external calcium concentration ([Ca^2+^]_ext_)-induced oscillation of [Ca^2+^]_cyt_(Cui *et al.* 2009). Further, although average Crz1 nuclear localization increases when [Ca^2+^]_ext_ increases(Cai *et al.* 2008), [Ca^2+^]_cyt_ is known to be under tight homeostatic control: the average [Ca^2+^]_cyt_ remains similar under a wide range of [Ca^2+^]_ext_(Miseta *et al.* 1999; Cui and Kaandorp 2006). Variation in average [Ca^2+^]_cyt_ is unlikely to follow the frequency of Crz1 pulsatility, which increases when [Ca^2+^]_ext_ increases(Cai *et al.* 2008). Thus, the relationship between calcium and Crz1 pulses remains unclear.

In this study, we examined the connection between [Ca^2+^]_cyt_ and Crz1 localization dynamics through dual fluorescence time-lapse microscopy(Lin *et al.* 2015). We found that, as recently reported (Carbó *et al.* 2017), cytosolic calcium concentration varies stochastically at the single cell level, showing bursts on the timescale of 10-100 sec. We observed overshoots of the calcium concentration, strongly implicating calcium channels in these bursts. We found that Crz1 pulses tend to follow these calcium bursts, but that the relationship is not simple: multiple Crz1 pulses may follow each calcium burst, and the number of Crz1 pulses depends on the size of the calcium burst. We modulated calcium channel activity and found much larger calcium bursts, which led to higher numbers of Crz1 pulses. To explain how [Ca^2+^]_cyt_ affects Crz1 nuclear localization, we developed a stochastic model of Crz1 nuclear localization and tested predictions in the experimental data. In general, stochastic pulses in signaling dynamics may be generated by time-delayed responses to fluctuations in second messenger concentration.

## Materials and methods

### Yeast cell strain and growth conditions

BY4741 was used to construct the dual Crz1-Calcium reporter strain. Plasmids expressing GCaMP3 calcium reporter were constructed using Gibson assembly protocol(Gibson 2011) and gel purification. The calcium reporter gene was assembled between the promoter of ribosomal protein L39, RPL39, and the ADH1 terminator. pRPL39-GCaMP3-tADH1 was integrated at the *HO* locus using a selectable marker (LEU2) and confirmed by Sanger sequencing. Four replicates were performed and all showed expected GCaMP3 expression (Tian *et al.* 2009 p. 3; Carbó *et al.* 2017). To tag Crz1 with mCherry at the C terminus, genomic integration of pCrz1-ymCherry was done at the *CRZ1* locus using a selectable marker (URA3) and confirmed by PCR. All transformations were performed using the standard lithium acetate procedure(Schiestl and Gietz 1989).

All the time-lapse imaging experiments were started when cells were in log-phase (4 hr after being diluted from overnight liquid culture). Cells were grown in synthetic complete (SC) media lacking leucine and uracil to maintain section of markers. Carbon source was 2% glucose. For artificially increased calcium burst experiments, 200µm Nifedipine were added during the 4 hr inoculation.

### Spinning-disk Confocal Microscopy and image analysis

Nikon CSU-X1 was utilized for time-lapse imaging at room temperature (22°). For GCaMP3, 488 nm laser was applied with time resolutions of 6 sec/frame, exposure time of 100 msec, and 25% laser intensity; for mCherry, 561 nm laser was applied with time resolution 30 sec/frame, exposure time of 700 msec, and 50% laser intensity. Bright field images with out-of-focus black cell edge were acquired every minute for cell segmentation and tracking.

Cells were attached to glass-bottom dishes with 0.1 mg/ml Concanavalin-A as a binding agent using a standard protocol (Pemberton 2014; Zarin *et al.* 2017). For each experiment, a time-lapse image series without calcium stress induction was recorded as a negative control. At the beginning of each time-lapse image series, an area of the dish that had not been exposed to laser was recorded in order to avoid blue light stress, which is known to induce Crz1 nuclear localization (Bodvard *et al.* 2013 p. 1). Calcium chloride solution was added to the dish to a final concentration of 0.2M through a syringe within 20 sec. To record the dynamics during steady state, time-lapse movies of 30 min or 1 hr were recorded after more than 1 hr of calcium stress induction for two to four time-lapse movies in each experiment. 27 replicates of time-lapse movies (18 hr in total) were recorded. Every analysis was done in both 1 hr and 30 min time-lapse experiments.

Segmentation was automatically performed by identifying the area within cell edge through MATLAB Image Segmentation Toolbox, and cell tracking was performed by identifying 90% overlapping cell areas between two time frames. Mis-segmented and mis-stracked objects were manually removed. 23-87 cells were identified in each time-lapse movie. Single cell photobleaching correction was conducted after single cell reporter intensities were quantified (see below) using bi-exponential regression (Vicente *et al.* 2007): for GCaMP3 intensity, correction was performed according to baseline intensity; for Crz1 expected nuclear signal, correction was performed according to Crz1 expected cytoplasmic signal. Baseline was normalized to 0 after photobleaching correction.

### Osmotic shock reduction

Crz1 localizes into the nucleus for 10 to 15 min after an osmotic shock (Denis and Cyert 2002). In the experiments where we artificially increased calcium bursts (nifedipine treatment), the effect from osmotic shock was undesirable because the calcium bursts occur immediately after addition of calcium. Change in osmotic pressure due to 0.2M calcium chloride is around 3.4 Pa, so prior to the experiment, sodium chloride solution was added (to reach 0.4M) to increase osmotic pressure to 6.1 Pa. When calcium chloride solution was added (so final concentrations of both sodium chloride and calcium chloride were 0.2M), the final osmotic pressure was now around 6.5 Pa, reducing the change in osmotic pressure before and after addition of calcium to around 0.5 Pa.

### Reporter intensity quantification

GCaMP3 intensity for each time point was estimated as average pixel intensity for all pixels in the cell.

Nuclear localization for each time point was quantified by fitting a mixture of a Gaussian distribution and a uniform distribution, and the parameters of distributions were estimated using expectation-maximization on the pixel data from each cell (see supplementary text for more details and derivation of the algorithm).

### Peak finding, pulse analyses and periodicity

Local maxima/minima were identified with Matlab function findpeaks. To smooth fluctuations shorter than 4 time points, Savitzky-Golay filtering was applied on each trajectory before defining the Crz1 pulse threshold, identifying Crz1 pulses, and quantifying calcium overshoot depth.

To define the threshold for calcium bursts and Crz1 pulses, every local maximum in all cells growing in standard liquid culture (no additional calcium) was identified with a minimum distance of 60 sec. Thresholds were then chosen to filter out most of the background noise: we chose the top 0.5% of the peak height (0.09) for calcium bursts, and the top 5% of both the peak height (0.30) and prominence (0.15) for Crz1 pulses.

For the analysis of the relationship between calcium burst height and number of Crz1 pulses, the Crz1 pulses following a calcium burst were counted until the next calcium burst or the end of the time series, and the Crz1 pulses before a calcium burst were counted until the previous calcium burst or the beginning of the time series.

For every cell that has its largest calcium burst after 5 min from the beginning or before 5 min from the end of the time-lapse experiments, its Crz1 trajectory was separated into pre-calcium-burst and post-calcium-burst trajectories. Each trajectory was evaluated with an aperiodic Gaussian process model or a periodic Gaussian process model by computing the log likelihood ratio (LLR) using an established method and MATLAB scripts (Phillips *et al.* 2017).

### Logistic pulse fitting

A least squares method was developed to quantify Crz1 pulse height and width based on the analytic solution of the logistic curve (see supplementary text for more details and derivation).

### Data availability

Strains are available upon request. The authors state that all data necessary for confirming the conclusions presented in the article are represented fully within the article. Supplemental material available at Figshare: https://doi.org/10.25387/g3.7485575.

## Results

### Calcium bursts are observed when yeast cells are under calcium stress

**Figure 1:**
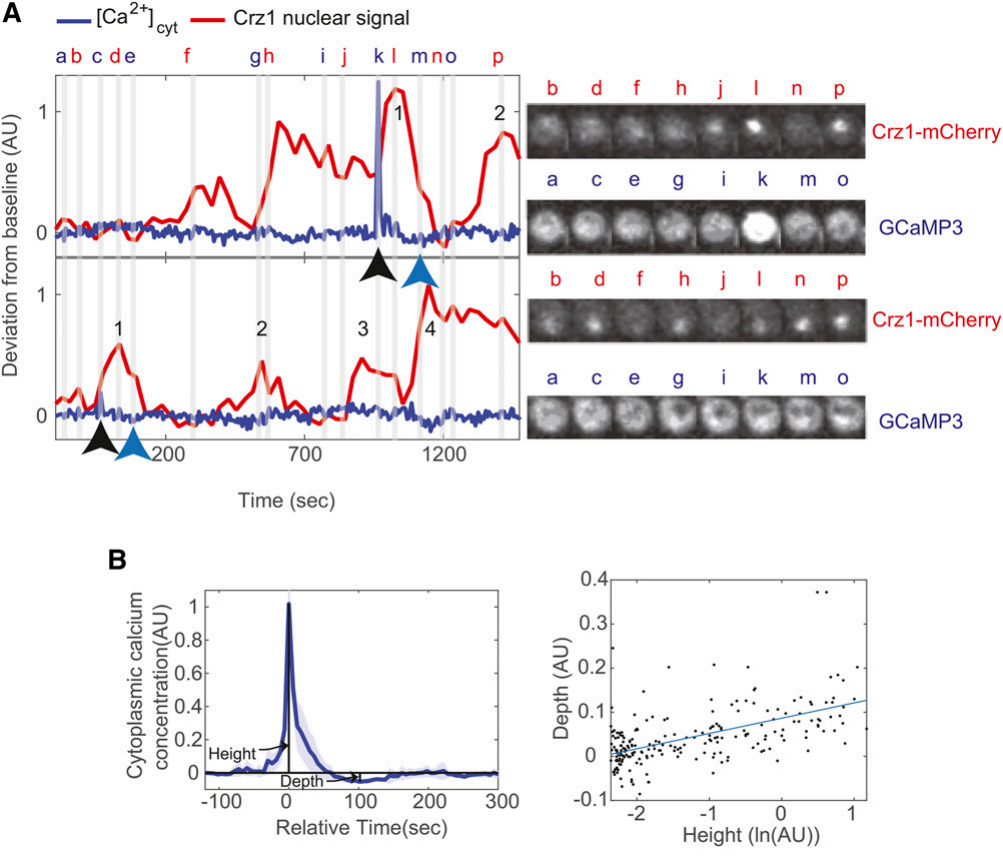
Calcium bursts with overshoots are found in yeast cells under calcium stress. A) The left panel shows examples of single cell trajectories for Crz1 (red trace) and calcium (blue trace) and snapshots at representative time points from two cells close to each other in the original field of view (grey lines and letters a∼p). Numbers along the red trace indicate the Crz1 pulses identified following a calcium burst (black arrow below blue trace). Blue arrow indicates the local minimum (so-called overshoot) following the calcium burst. Images in the right panel show the mCherry channel and GCaMP3 channel at points indicated in the left panel. B) The left panel shows the average trace of 50 calcium bursts. The shaded area shows 95% confidence interval (based on a normal distribution) of the average trace. Maxima of calcium bursts are aligned to time = 0 sec (Relative time). Overshoot can be found around time = 100 sec. In the right panel, each dot represents a single calcium burst. The x-axis is in natural log of peak height, while the y-axis is the depth of the overshoot. The blue line shows a linear fit (R^2^ = 0.37).

In order to study the relationship between the dynamics of [Ca^2+^]_cyt_ and Crz1 nuclear localization, we constructed a dual Crz1-Calcium reporter strain and measured dynamics using time-lapse microscopy. We tagged Crz1 with the mCherry fluorescent protein in a strain with a cytoplasmicly expressed calcium sensor, GCaMP3 (Tian *et al.* 2009) and use this as an indicator of cytosolic calcium concentration. After binding to calcium ions, GCaMP3 undergoes a conformational change and emits green fluorescent light. We then recorded movies on a confocal fluorescence microscope while cells were under calcium stress (see Methods). As in a previous study(Cai *et al.* 2008), we observed stochastic and rapid increases and decreases of [Ca^2+^]_cyt_ when yeast are under calcium stress (Figure 1 A, supplementary video 1). We noticed that these “calcium bursts” (defined by a threshold ratio above background, see Methods) are followed by an overshoot of calcium concentration below the resting level (average of 50 largest calcium local maxima in a representative time-lapse movie is shown in Figure 1B left panel), whose depth is positively correlated to the height of the pulse (Figure 1B, right panel, R^2^= 0.37). This overshoot cannot fit an exponential curve and suggests negative feedback on [Ca^2+^]_cyt_, which is consistent with the predictions of calcium models constructed in previous studies of homeostasis(Ni *et al.* 2009; Thorsen *et al.* 2014).

### Stochastic Crz1 nuclear localization pulses follow calcium bursts at steady state

**Figure 2:**
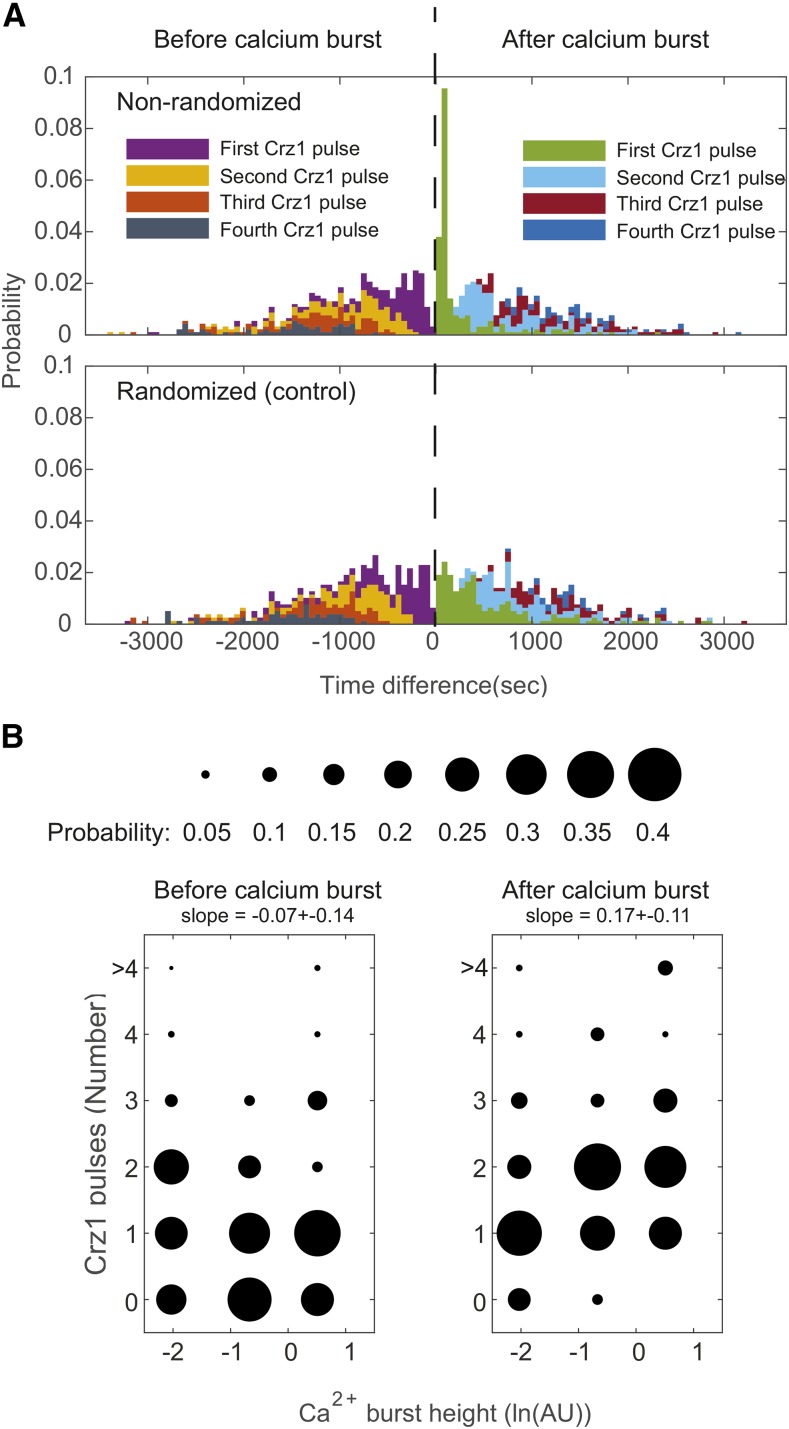
A calcium burst is followed by multiple Crz1 pulses, and the number of Crz1 pulses is positively correlated to the height of the calcium burst. A) Upper stacked histogram presents the probability of first, second third and fourth Crz1 pulses plotted as a function of the time they occur relative to calcium bursts from the same cells (black dashed line). Crz1 pulses found before calcium bursts (purple, yellow, orange, gray) are plotted on the left side of the dashed line with the negative time difference. Crz1 pulses found after calcium bursts (green, cyan, red, and blue) are plotted on the right side of the dashed line with the positive time difference. The lower stacked histogram shows the same analysis from randomly chosen cells. B) Data are divided into three groups based on the height of calcium bursts (low, medium, and high calcium bursts, n = 83, 29, 23) and aligned to the mean calcium burst heights of each group. The size of dots represents the probability of finding a number of Crz1 pulses in a group.

Individual cell trajectories do not show a simple relationship between [Ca^2+^]_cyt_ and Crz1 nuclear localization (Figure 1A), so we next sought to understand how Crz1 pulses (see Methods for definition of Crz1 pulses) are affected by calcium bursts. We analyzed the distribution of the time differences between a calcium burst and following Crz1 pulse(s) (using so-called pulse-triggered averaging (Lin *et al.* 2015)). The coherence of the first and second pulses suggested to us that one or more Crz1 pulses follow a single calcium burst (Figure 2A). We therefore compared the time until the first Crz1 pulse of a calcium burst to that from a randomly chosen cell (that may or may not show a calcium burst.) Consistent with our hypothesis, we found that the time until the first Crz1 pulse after a calcium burst shows reduced standard deviation (389.02 sec *vs.* 586.64 sec, F-test, *P* < 10^−7^, n = 193, 169, Bonferroni correction, α = 0.0031) and occurs sooner than observed in randomly chosen cells (251.42 sec *vs.* 586.64 sec for random, two-tailed *t*-test, *P* < 10^−8^, n = 193, 169, Bonferroni correction, α = 0.0031). A similar range of time differences is also observed in cross-correlation analysis (supplementary figure 1). In contrast, the distribution of the first Crz1 pulse before a calcium burst is not different from that of randomly chosen cells (average differences are -542.87 sec *vs.* -579.10 sec for random, two-tailed *t*-test, *P* > 0.5; standard deviations are 516.98 sec *vs.*551.17 sec for random, F-test, *P* > 0.4, n = 168,155), suggesting that the occurrence of Crz1 pulses before calcium bursts is independent of calcium bursts and consistent with the idea that calcium bursts cause Crz1 pulses.

### Multiple Crz1 pulses are associated with each calcium burst

To understand if Crz1 pulses following the first Crz1 pulses are also associated with calcium bursts, we again compared the time difference between Crz1 pulses to that from randomly chosen cells (that may or may not show a calcium burst). We found that, although the mean time differences between first and second Crz1 pulses and that between second and third Crz1 pulses are not significantly shorter, the standard deviation of time differences is significantly smaller (F-test for the second Crz1 pulse *vs.* random, 384.88 sec *vs.* 649.91 sec, *P* < 10^−5^, n = 138, 113; F-test for the third Crz1 pulse *vs.* random, 336.79 sec *vs.* 626.08 sec, *P* < 0.003, n = 96, 69, Bonferroni correction, α = 0.0031; Table S1 contains all the statistical summaries of pulse comparison). This increased coherence suggests that one calcium burst can lead to multiple Crz1 pulses.

### A correlation between calcium burst height and Crz1 pulse number suggests a noisy analog-to-digital converter

We next sought to identify factors that determine the number of Crz1 pulses that follow each calcium burst. Because the length of time between two stochastic calcium bursts (‘interval’) is random, short intervals might preclude observation of all generated Crz1 pulses. In order to reduce this effect, we filtered out calcium bursts where the interval following was less than 10 min. We found that the number of Crz1 pulses after calcium bursts is positively correlated to the height of calcium bursts (Figure 2B, generalized linear model regression with Poisson distribution, slope = 0.17+-0.11, *P* = 0.0016; supplementary figure 2 presents analysis with standard linear model regression). We also divided the data into three groups based on the height of calcium bursts (low, medium, and high calcium bursts, n = 83, 29, 23), and found that the average number of Crz1 pulses following large calcium bursts is significantly larger than that of small calcium bursts (two-tailed *t*-test, *P* = 0.0011). To test whether a calcium burst causes Crz1 pulses, we tested whether there is a correlation between calcium burst height and the number of Crz1 pulses before calcium bursts. If an (unmeasured) third factor affects both the height of a calcium burst and the number of the Crz1 pulses in a cell, we expect more Crz1 pulses both before and after large calcium bursts. We found that the number before is not correlated with calcium burst height (slope = -0.07+-0.14, *P* > 0.3, generalized linear model regression with Poisson distribution, Figure 2B). Finally, to control for the effect of the interval (as discussed above), we used the residuals of a regression of Crz1 pulse number on interval length as a measure of Crz1 pulse number independent of the interval. The residual of this regression after, but not before calcium bursts was significantly correlated with the calcium burst size (supplementary figure 2, slope = 0.22, *P* = 0.005 *vs.* slope = -0.11, *P* = 0.17). These results are consistent with the idea that calcium burst heights are converted into digital numbers of Crz1 pulses, albeit through a noisy process.

### Artificially increasing calcium burst size supports the noisy analog-to-digital converter analogy

**Figure 3:**
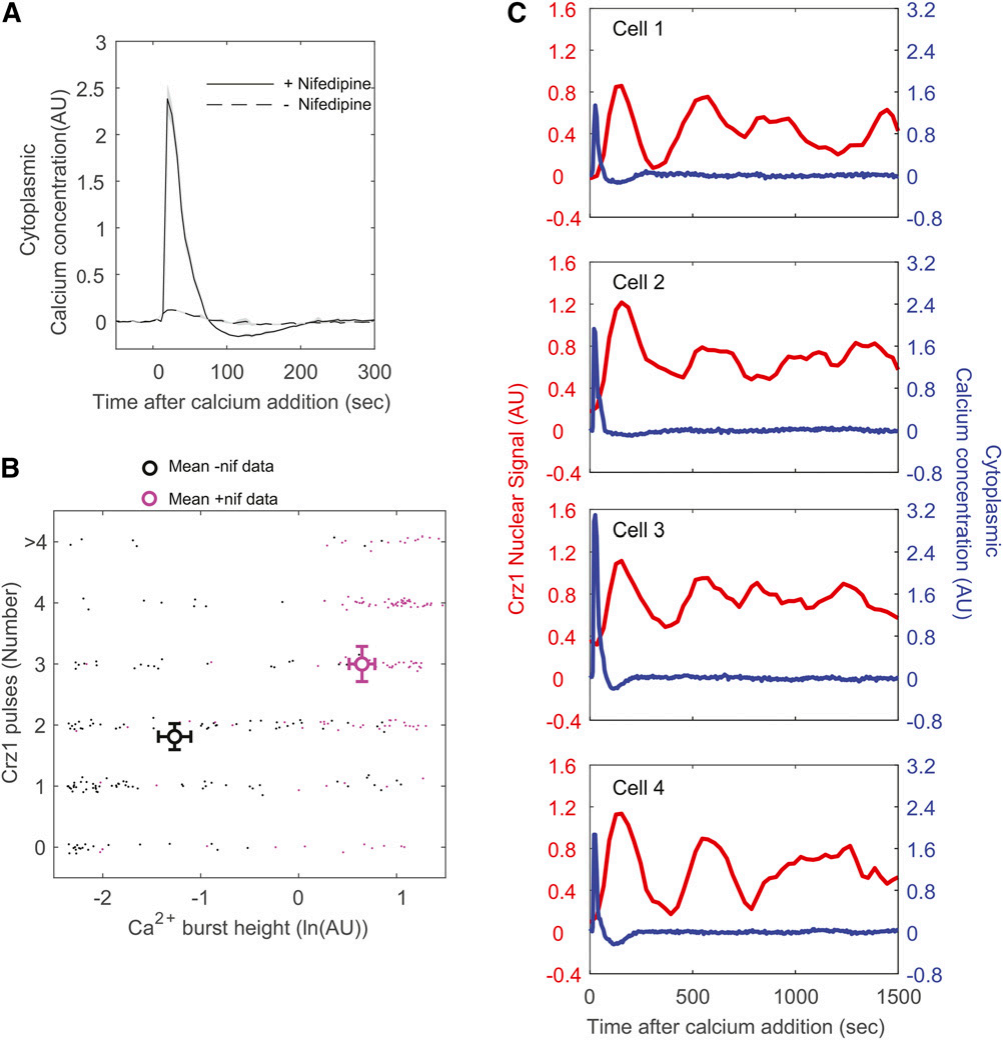
Artificially induced calcium bursts with large average height are followed by more Crz1 pulses. A) Average single cell trajectory (without aligning the maxima) after calcium addition shows synchronized calcium bursts in cells treated with nifedipine (solid line is mean GCaMP3 signal), but not in untreated cells (broken line is the mean GCaMP3 from untreated cells. Shaded areas show the 95% CI of mean (based on the normal distribution). B) Average Crz1 pulses number against average calcium burst height shows higher number and height from nifedipine treated cells (magenta circle). Error bars represent 95% CI of mean (based on the normal distribution on the x-axis and Poisson distribution on the y-axis). Dots represent each calcium burst after which the Crz1 pulses were counted and the height of the burst. Since the Crz1 pulses are integers, a small random number is introduced separate dots on the y-axis (“jitter”). C) Four single cell examples of Crz1 nuclear localization dynamics (red lines) and cytoplasmic calcium dynamics (blue lines) from nifedipine treated cells.

The analogy of a noisy analog-to-digital converter between calcium burst height and Crz1 pulse number predicts that the average number of Crz1 pulses following calcium bursts could be made larger by artificially inducing higher calcium bursts. To test this prediction, we treated inoculated cells with nifedipine (See Methods), an ion channel blocker that has been shown to partially inhibit the activity of a membrane calcium channel, Cch1-Mid1(Teng *et al.* 2008), and may affect the occurrence of calcium bursts. By doing so, we reliably induced synchronized calcium bursts (supplementary video 2) that were on average twice as large as the stochastic pulses observed at steady state in 0.2M calcium treatment alone (Figure 3A, compared to Figure 1B). We also utilized an ion-exchange method that maintains osmotic pressure during calcium treatment (see Methods and supplementary figure 3) to reduce the effect of osmotic shock on [Ca^2+^]_cyt_, because previous studies show that under osmotic shock vacuolar calcium ions are released into cytoplasm through a vacuolar calcium channel, Yvc1 (Denis and Cyert 2002). As predicted by the noisy analog-to-digital converter, the significantly larger calcium bursts are associated with significantly more Crz1 pulses (Figure 3B, mean log height = -1.27+-0.17 *vs.* 0.65+-0.13, two-tailed *t*-test, *P* < 10^−47^ ; mean pulse number = 1.81+-0.21 *vs.* 3.00+-0.29, two-tailed *t*-test, *P* < 10^−11^, n = 139 *vs.* 153). In individual cells, these large calcium bursts are typically followed by at least three Crz1 pulses, and Crz1 dynamics now clearly appear to oscillate while no oscillations are observed in calcium concentration (Figure 3C). These results strongly support our claim that single calcium bursts are followed by multiple Crz1 pulses, and are consistent with the analogy that a noisy analog-to-digital converter in the calcium/calcineurin signaling pathway converts calcium burst height into Crz1 pulse number.

### A simple time delay model can reproduce the properties of Crz1 pulses after calcium bursts

**Figure 4:**
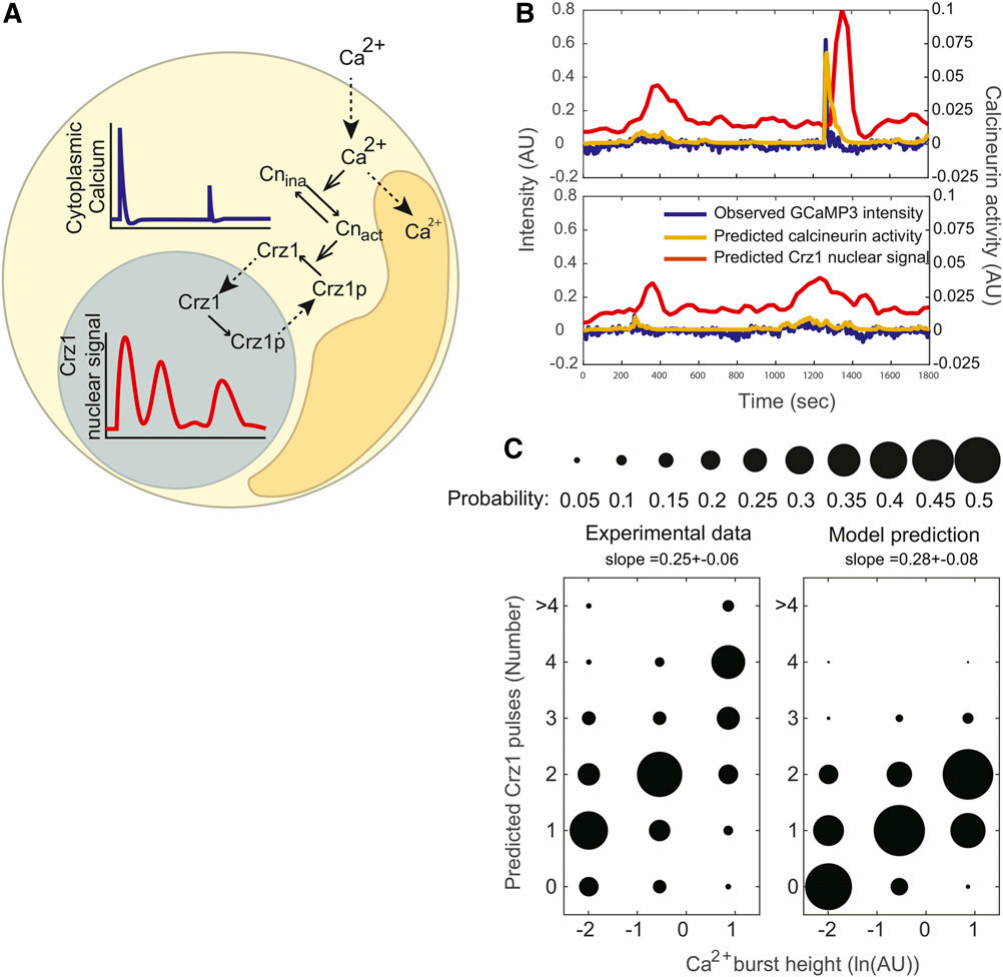
A time delay model for Crz1 nuclear pulsing can qualitatively reproduce Crz1 dynamics after calcium bursts. A) In this single cell model, Ca^2+^ in the cytoplasm (yellow shaded area) is controlled through a two-channel system (dotted arrows crossing from outside the cell to inside, and crossing from yellow shaded area to orange shaded area representing the vacuole). Calcineurin is activated (Cn_ina_ to Cn_act_) by cytoplasmic calcium and leads to Crz1 dephosphorylation (Crz1p to Crz1). Dephosphorylated Crz1 imported (dotted arrow) from the cytoplasm (yellow shaded area) to nucleus (gray shaded area) and exported (dotted arrow from the nucleus to cytoplasm). B) The Crz1 pulses produced by the model (red trace) following calcium bursts (blue trace). The gold trace shows the calcineurin activity predicted by the model. C) Right panel summarizes the model prediction of Crz1 pulses based on the experimental calcium trajectories (steady state and nifedipine treated trajectories combined). Data are divided into three groups based on the height of calcium bursts (low calcium bursts, medium calcium bursts, and high calcium bursts, n = 101, 48, 142) and plotted according to the mean calcium burst heights of each group. The size of dots represents the probability of finding a number of Crz1 pulses in a group. The left panel shows the same summary of the experimentally observed Crz1 pulses.

To explain the mechanistic connection between calcium bursts and Crz1 pulses, we considered a two-step process in single cells (Figure 4A). The first step is that external calcium concentration leads to cytosolic calcium bursts (4A, blue trace) through stochastic channel opening, and the second step is that a calcium burst leads to nuclear Crz1 pulses (4A, red trace) through the calcineurin pathway.

A negative feedback loop in the calcineurin pathway could lead to oscillation of calcineurin activity and drive Crz1 pulses, but we decided not to include one in our model for two reasons. First, the known negative feedback loop in the calcineurin pathway through Rcn1 does not appear to affect Crz1 pulsatility. Rcn1 is an inhibitor of calcineurin that is degraded when phosphorylated and is dephosphorylated by activated calcineurin (Kishi *et al.* 2007; Rodríguez *et al.* 2009), thus leading to negative feedback. However, the negative feedback loop is thought to be controlled by the protein abundance of Rcn1, as phosphorylation does not prevent Rcn1’s inhibition of calcineurin(Kishi *et al.* 2007). Since Crz1 pulsatility occurs when protein synthesis is inhibited by cycloheximide (supplementary figure 4) we consider it unlikely that Rcn1 provides negative feedback through changes in protein abundance. Second, models with feedback mechanisms assume that the amplitude of Crz1 pulses is a sensitive readout of calcineurin activity though calcineurin binding, and therefore predict increased Crz1 pulse amplitude when the affinity of the calcineurin docking site on Crz1 is increased (Cai *et al.* 2008). In contrast, the Crz1 pulse frequency, but not amplitude increases when the affinity of the calcineurin docking site on Crz1 is increased (Cai *et al.* 2008). Therefore, we worked toward models that do not include a feedback mechanism.

Previously, Crz1 nuclear localization dynamics were explained with a conformational switch model (Cui and Kaandorp 2006). This model assumes that the large number of phosphorylation sites on Crz1 leads to a sigmoid function relating calcineurin activity to Crz1 nuclear localization, so when calcineurin activity swings above and below a threshold, Crz1 sensitively reads out the perturbation in calcineurin activity and switches fully nuclear or cytoplasmic (Salazar and Höfer 2003; Cui and Kaandorp 2006). This model would predict that calcium concentration crosses a threshold before each Crz1 pulse, and pulses stop once calcium oscillations decay below the threshold. However, calcium is not observed to pass a threshold before each Crz1 pulse in our data (*e.g.*, Figure 3B).

We therefore considered another single cell model. Inspired by the observations on the population level that Crz1 pulses tend to occur within 100 sec after calcium bursts and then disperse over time, and that higher calcium bursts lead to more Crz1 pulses, we constructed a discrete-time stochastic model that explains single cell Crz1 nuclear localization based on time delays during nuclear import and export with variation among Crz1 molecules. Time delay models have been constructed through different approaches, including deterministic and stochastic delay differential equations with a fixed or variable delay periods (Mittler *et al.* 1998; Bratsun *et al.* 2005; Galla 2009; Longo *et al.* 2013). Here, we used a discrete-time Markov chain because it is simple to simulate trajectories. In the model, Crz1 molecules transit between the nucleus and the cytoplasm in a coordinated manner (show pulsing dynamics on average) only when calcineurin activity is very high to a recent calcium burst. As calcineurin activity slowly returns to its basal level, the coordinated transport of 500 Crz1 molecules in a single cell decoheres.

We model the Crz1 nuclear signal by aggregating the states of individual Crz1 molecules after an increase in cytosolic calcium concentration of a single cell. As the input to the model, we provide calcium concentration, *Ca*, as a function of time, *t*, which can be obtained from experimental data (using GCaMP reporter fluorescence as a proxy). We note that, although calcium concentration can never be negative, *Ca* can be negative because our GCaMP reporter data are normalized such that baseline fluorescence level is defined as 0. When calcium bursts overshoot, we obtain negative values.

We assume that calcineurin activity at time *t*, *Cn*(*t*), has an activation rate proportional to calcium concentration and a constant rate of decay. The discrete time dynamics of *Cn*(*t*) is described by

$$\Delta Cn\equiv Cn\left(t+1\right)-Cn\left(\text{t}\right)=\left(Cn_{base}-Cn\left(t\right)\right)D+\max\left[0,Ca\left(t\right)\right]A,$$

where $Cn_{base}$ is the basal activity of calcineurin, D is the decay rate of calcineurin activity, and A is the activation rate by cytosolic calcium. The probability of a Crz1 molecule being imported is *Cn*(*t*) multiplied by the probability of dephosphorylation by an active calcineurin molecule (see Methods for details). Thus, when calcineurin activity increases, the probability of a Crz1 molecular being imported increases.

Once a Crz1 molecule is imported into the nucleus, it returns to the cytoplasm after it is phosphorylated in the nucleus, which we assume occurs at a constant rate. These chemical reactions can be formulated using a standard biochemical rate approach as

$$X_{C}\overset{a}{\Rightarrow }X_{N},\;\;X_{N}\overset{b}{\Rightarrow }X_{C},\;$$

where $X_{C}$ and $X_{N}$ are cytoplasmic and nuclear Crz1 molecules, respectively, and a and *b* are the rates of delayed transports (denoted as thick arrows). In our Markov chain framework, we assume that transports are multistage, so the delay time follows a Gamma distribution with the two parameters defined as the number of states and the transition probability (see Supplementary text for details).

This time-delay model can qualitatively reproduce Crz1 pulses after calcium bursts (Figure 4B). To understand if the model can also qualitatively reproduce the noisy analog-to-digital conversion behavior, we used the model to predict the Crz1 trajectories of our single cells based on their measured calcium trajectories. Although it is not clear whether an identical mechanism generated both the Crz1 pulses during steady state and under nifedipine treatment, we pooled the data in order to widen the range of calcium burst heights. The predictions show that the number of Crz1 pulses after a calcium burst is positively correlated to the height of that calcium burst, with a slope comparable to that of experimental data (Figure 4C, generalized linear model regression with Poisson distribution, predicted slope = 0.28+-0.08, *P* < 10^−10^, experimental slope = 0.25+-0.06, *P* < 10^−15^). These results suggest that the time delay model can explain the noisy analog-to-digital converter.

### Other predictions of the model are found in the experimental data

**Figure 5:**
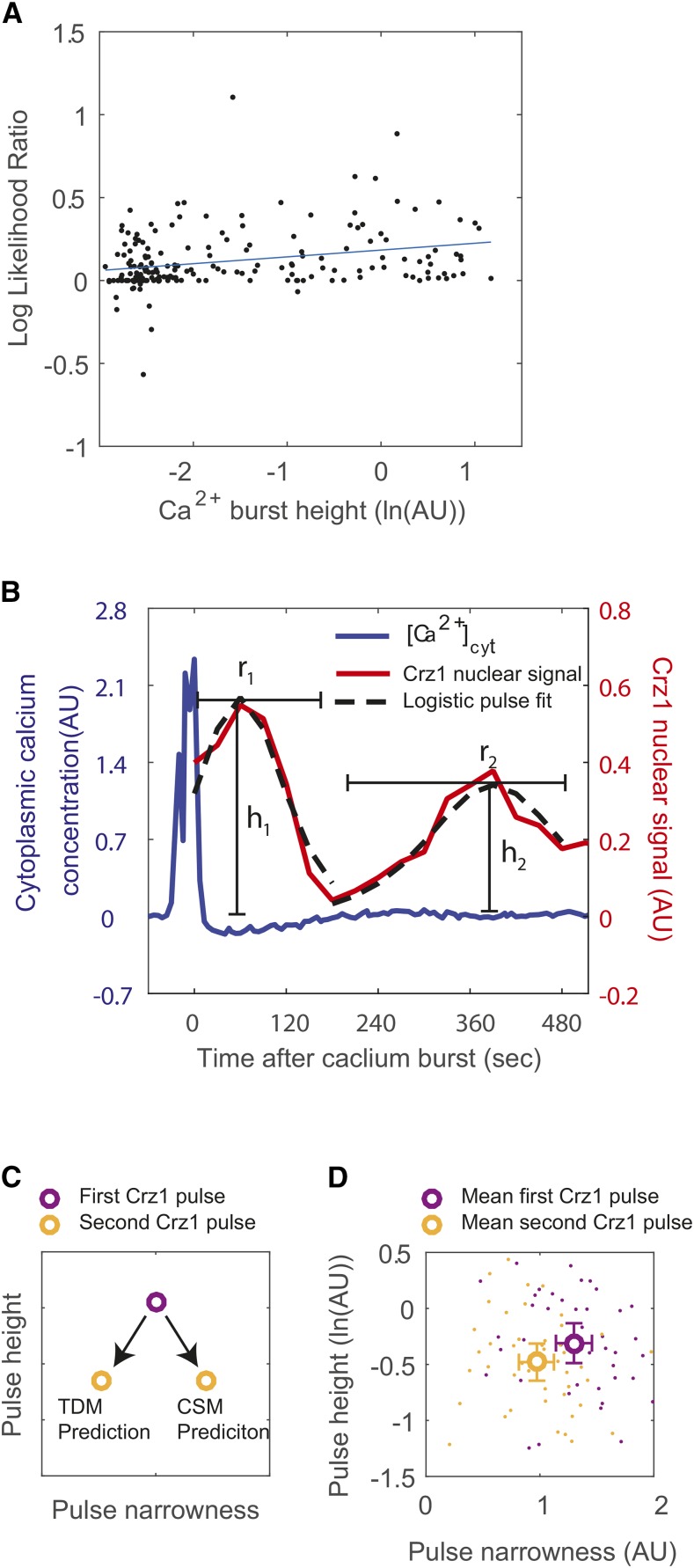
Predictions of the time delay model are confirmed by experimental data. A) Log-likelihood ratio between a periodic and an aperiodic Gaussian process model shows an increasing preference toward the periodic process when calcium burst height increases (blue line shows a linear fit, R^2^ = 0.07). Each dot represents the Crz1 trajectory following a calcium burst. B) shows an example of logistic pulse fit (dotted trace) to experimental data (red trace) for estimating the narrowness (r_1_ and r_2_) and the heights (h_1_ and h_2_) of the first and second Crz1 pulses after a calcium burst. C) Conformational switch model (CSM) predicts that the second Crz1 pulse is shorter and narrower than the first Crz1 pulse, while the time delay model (TDM) predicts that the second Crz1 pulse is shorter and less narrow than the first. D) Unfilled circles indicate the mean height and narrowness of Crz1 pulses. The first and second Crz1 pulses are purple and yellow, respectively. Each dot corresponds to a single Crz1 pulse identified following a calcium burst.

The time delay model also predicts other properties of Crz1 pulsatility. A first prediction is that the periodicity of a Crz1 trajectory is correlated with calcium burst size, such that a Crz1 trajectory after a higher calcium burst keeps oscillating longer. To test this prediction, we quantified the periodicity of Crz1 dynamics after the highest calcium burst for each cell using a Gaussian Process model (see Methods), which computes the log-likelihood ratio (LLR) comparing a periodic to an aperiodic kernel. The LLR of post-calcium-burst trajectories is correlated to the height of calcium bursts (Figure 5A, slope estimated with linear regression model is 0.050+-0.020, *P* = 0.019). Calcium bursts higher than 0.11 show LLR significantly larger than that of the rest (two-tailed *t*-tests, sample sizes are 92 and 95, *P* < 10^−5^). These results indicate that higher calcium bursts lead to Crz1 dynamics that can be better described by a periodic Gaussian process. As a control, we also computed the LLR for pre-calcium-burst trajectories and found that the pre-calcium-burst trajectories of for calcium burst height above 0.11 are not statistically more periodic (*P* > 0.1). Periodic dynamics after high calcium bursts would also be predicted by conformational switch model, where, after a higher calcium burst, calcium oscillates longer and has more peaks crossing a threshold to trigger Crz1 pulse (supplementary figure 5).

Additional predictions of the time delay model are that, for a calcium burst that is followed by at least two Crz1 pulses, the second Crz1 pulse is shorter and wider than the first Crz1 pulse because the coordinated transport of Crz1 molecules disperses across time. Although both the conformational switch model and the time delay model predict shorter second Crz1 pulses, the conformational switch model would predict a narrower second Crz1 pulse as the calcium oscillations decay (Figure 5C). To test these predictions, we identified the calcium bursts that are followed by two Crz1 pulses, and fit them to a “logistic pulse model” (Figure 5B, median R^2^ = 0.85, mean R^2^ = 0.79, See Methods) to estimate pulse height and narrowness. We found that the height of the second pulse is significantly smaller (Figure 5D, paired *t*-test, *P* < 0.005, n =42), and that the mean narrowness of the second pulses is significantly smaller than that of the first pulses (Figure 5D, paired *t*-test, *P* < 10^−8^, n = 42), supporting the time delay model to the exclusion of the conformational switch model.

Thus, the data support three additional predictions of a simple stochastic model of Crz1 nuclear import and export. Together with the explanation of the noisy analog-to-digital converter, our results support the idea that coordination of Crz1 localization (and thus pulsatility) is the result of a time-delay in nuclear import and export (see Discussion).

## Discussion

Our results suggest that the link between [Ca^2+^]_cyt_ and Crz1 pulses is analogous to a noisy analog-to-digital converter: higher calcium bursts lead to more Crz1 pulses. This provides a possible link between irregular calcium oscillation and transcription (Song *et al.* 2012). However, the correlation between calcium bursts and Crz1 pulse number at steady state is not strong. The strongest effect of calcium pulses at steady state is clearly to cause the first Crz1 pulse (Figure 2A), so the variation in Crz1 pulse number could be due in part to random Crz1 pulses (not caused by calcium bursts, discussed below) that happen to follow this first Crz1 pulse. In that case, we would still expect the observed correlation at steady state, but an additional explanation would be needed for the nifedipine treated cells, which are unambiguously showing greater than two Crz1 pulses (Figure 3C). Instead, we believe the weak correlation in steady state is due to a number of factors: first, the time interval after calcium bursts is random, and therefore we may simply not observe Crz1 pulses because another calcium burst occurs. Second, although we introduced methods to identify pulses in single cells and quantify calcium burst size in yeast, these methods are not perfect: differences in imaging conditions and cell morphology may lead to error in quantification and reduce the correlation.

Finally, as noted above, Crz1 fluctuations are also found without preceding calcium bursts. These Crz1 fluctuations are aperiodic and do not show correlations with calcium burst size. We recorded movies at four different [Ca^2+^]_ext_, and found that, although the frequency of calcium bursts is correlated with external calcium concentration, the increase in calcium pulsing frequency is not comparable to the increase in Crz1 pulsing frequency, and the average size of calcium bursts does not increase significantly (supplementary figure 6), so we cannot fully explain the calcium concentration dependence of Crz1 fluctuations. However, cross-correlation analysis shows that these Crz1 dynamics have only a small correlation with [Ca^2+^]_cyt_ dynamics (supplementary figure 1). We suggest that other intrinsic environmental fluctuations that affect Crz1 localization, such as light-(Bodvard *et al.* 2013), osmotic pressure-(Denis and Cyert 2002), or glucose-(Bouillet *et al.* 2012; D’hooge *et al.* 2015) induced Crz1 regulation might be involved in producing these fluctuations.

One of the interesting properties of pulsatile dynamics is that the pulses in individual cells are not synchronized, despite cells experiencing the same environmental stress (Dalal *et al.* 2014). We explain this aspect of Crz1 pulsatility by arguing that the [Ca^2+^]_cyt_ among individual cells at a given time point is stochastic, due to spontaneous calcium transients(Baker *et al.* 2016). Thus, if calcium bursts among cells could be synchronized in time, then Crz1 pulsatility should be synchronized immediately after, and that this induced synchrony would gradually decay. Consistent with this, in our nifedipine treated cells, where high calcium bursts were induced in every cell immediately after calcium was added into the media, the Crz1 pulses following these synchronized calcium bursts are also synchronized, and this synchrony decays with time (supplementary figure 3B). However, by simulating Crz1 dynamics many times with identical parameter values (Table S2), we found that our stochastic time delay model does not predict the loss of synchrony at the population level. This suggests that additional sources of cell-to-cell variability are likely missing from the stochastic time delay model.

Nevertheless, our stochastic time delay model has several advantages over a conformational switch model that assumes Crz1 nuclear localization sensitively reads out [Ca^2+^]_cyt_ when [Ca^2+^]_cyt_ passes through a threshold(Cui and Kaandorp 2006). Once a damped calcium oscillation is present, the conformational switch model can generate Crz1 pulses as a readout of [Ca^2+^]_cyt_ passing through a threshold (supplementary figure 5). Although we do not observe calcium oscillations passing a threshold in our movies, it is possible that our calcium sensor is not sensitive enough to distinguish these dynamics from background noise. Both our model and the conformational switch model require no negative feedback in the calmodulin/calcineurin signaling pathway. However, our model predicts the width of the second Crz1 pulse to be wider than the first, while the conformational switch model predicts the opposite: a narrower second Crz1 pulse because it is reading out a smaller fluctuation in [Ca^2+^]_cyt_. The comparison of pulse widths (Figure 5D) supports the time delay model. We also note that the stochastic model is simpler (fewer parameters needed to generate pulses and no assumption a sensitive threshold), and can directly explain the coordination of the subcellular localization of the ∼500 Crz1 molecules in the cell through time-delay in nuclear transport.

One crucial assumption in our model for the coordination among Crz1 molecules is the deactivation rate of calcineurin. Previous studies show that calcineurin has a deactivation rate *in vitro* of 0.08 fold change per min while both calcium ions and calmodulin are presented, and has an even slower deactivation rate when either of them is not presented(King and Huang 1984; Kincaid *et al.* 1986; Pallen and Wang 1986). The deactivation rate is slow enough to maintain the synchronous translocation of each Crz1 molecule in our model, which only requires calcineurin to return to baseline activity around 5 min after a calcium burst, a length of time that has been reported *in vitro*(King and Huang 1984; Kincaid *et al.* 1986; Pallen and Wang 1986). A conclusive test of our model would be a mutation in calcineurin that solely affects the deactivation rate, but, to our knowledge, no such mutant is available. Finally, we emphasize that our time delay model does not presume an analog-to-digital conversion mechanism, but instead explains multiple Crz1 pulses as a result of coordinated molecular movement. The ambiguity (discussed above) in the analog-to-digital converter analogy is irrelevant to the support of the data for our time delay model.

Previous work on Crz1 pulsatility suggested that Crz1 pulses are actively generated rather than passively reading out the fluctuation in [Ca^2+^]_cyt_(Cai *et al.* 2008). If our model is correct, then it suggests a third possibility: individual Crz1 molecules read out [Ca^2+^]_cyt_ with a time delay. This possibility can explain the observation that higher affinity of calcineurin docking site on Crz1 leads to higher pulsing frequency(Cai *et al.* 2008), because higher affinity allows Crz1 to be dephosphorylated by a lower fraction of activated calcineurin and, therefore, oscillate longer after a calcium burst (supplementary figure 7). The time delay is assumed to be created by the transport between cytoplasm and nucleus, which because it requires a complicated series of steps, leads to a transport rate in the order of minutes(Ribbeck and Görlich 2001). This model can be generalized to relocalization of other pulsatile transcription factors and macromolecules that have dynamics on the order of minutes, and can explain how signals that are short and fluctuating are converted into the frequency of pulses without a negative feedback loop.
